# The outcome‐representation learning model of decision‐making capacity in mild cognitive impairment

**DOI:** 10.1002/alz.092475

**Published:** 2025-01-03

**Authors:** Mang Zhang, Ying Zhang, Yuhan Xie, Yaonan Zheng, Luchun Wang, Huizi Li, Xin Yu, Huali Wang

**Affiliations:** ^1^ Peking University, Beijing, Beijing China; ^2^ Beijing Anding Hospital, Beijing, Beijing China; ^3^ Peking University Institute of Mental Health (Sixth Hospital), Beijing China; ^4^ Peking University, Beijing China; ^5^ National Clinical Research Center for Mental Disorders, Beijing China

## Abstract

**Background:**

The decision‐making capacity of persons with mild cognitive impairment (MCI) has not been fully explored. This study aimed to examine the decision‐making capacity in MCI using the outcome‐representation learning model.

**Method:**

52 persons with MCI and 49 healthy controls were recruited in the study. The Iowa Gambling Task (IGT) was administered to measure decision‐making under ambiguity situations. Five parameters, including the reward learning rate, the punishment learning rate, forgetfulness, win perseverance and deck perseverance, were included in the outcome‐representation learning (ORL) model to capture changes in decision‐making abilities in MCI patients.

**Result:**

Individuals with MCI exhibited reduced learning from losses outcomes and similar learning from wins outcomes compared to healthy controls. Additionally, MCI individuals exhibited less susceptibility to forgetfulness in the IGT compared to controls, suggesting that memory fading of choices made by MCI patients was not improved. Although MCI individuals still favored high‐win frequency decks like controls, their preference appeared to dimmish from the first to second completion of the IGT.

**Conclusion:**

The findings suggest that individuals with mild cognitive impairment have difficulty in learning lessons from decision‐making failures in complex contexts. It offers a potential novel computational neurocognitive marker for mild cognitive impairment.

